# A framework for multi-sensor satellite data to evaluate crop production losses: the case study of 2022 Pakistan floods

**DOI:** 10.1038/s41598-023-30347-y

**Published:** 2023-03-14

**Authors:** Faisal Mueen Qamer, Sawaid Abbas, Bashir Ahmad, Abid Hussain, Aneel Salman, Sher Muhammad, Muhammad Nawaz, Sravan Shrestha, Bilal Iqbal, Sunil Thapa

**Affiliations:** 1 International Centre for Integrated Mountain Development (ICIMOD), Islamabad, Pakistan; 2 Smart Sensing for Climate and Development, Center for Geographical Information System, University of the Punjab, Lahore, Pakistan; 3 Department of Land Surveying and Geo-Informatics, The Hong Kong Polytechnic University, Kowloon, Hong Kong; 4 Pakistan Agricultural Research Council (PARC), Islamabad, Pakistan; 5 Islamabad Policy Research Institute (IPRI), Islamabad, Pakistan

**Keywords:** Climate sciences, Environmental sciences, Climate-change impacts

## Abstract

In August 2022, one of the most severe floods in the history of Pakistan was triggered due to the exceptionally high monsoon rainfall. It has affected ~ 33 million people across the country. The agricultural losses in the most productive Indus plains aggravated the risk of food insecurity in the country. As part of the loss and damage (L&D) assessment methodologies, we developed an approach for evaluating crop-specific post-disaster production losses based on multi-sensor satellite data. An integrated assessment was performed using various indicators derived from pre- and post-flood images of Sentinel-1 (flood extent mapping), Sentinel-2 (crop cover), and GPM (rainfall intensity measurements) to evaluate crop-specific losses. The results showed that 2.5 million ha (18% of Sindh’s total area) was inundated out of which 1.1 million ha was cropland. The remainder of crop damage came from the extreme rainfall downpour, flash floods and management deficiencies. Thus approximately 57% (2.8 million ha) of the cropland was affected out of the 4.9 million ha of agricultural area in Sindh. The analysis indicated expected production losses of 88% (3.1 million bales), 80% (1.8 million tons), and 61% (10.5 million tons) for cotton, rice, and sugarcane. This assessment provided useful tools to evaluate the L&D of agricultural production and to develop evidence-based policies enabling post-flood recovery, rehabilitation of people and restoration of livelihood.

## Introduction

There is increasing scientific evidence that human-induced climate change has caused severe environmental impacts and jeopardised global food security^1–3^. The frequency of floods and other extreme events has increased recently in South Asia and worldwide^4^ causing significant loss and damage, particularly in Pakistan^5^. Since the independence of Pakistan in 1947, the recurrence of the major flood is almost a decade or less, mostly due to heavy monsoon rainfall in July and August causing severe losses in terms of human lives, agriculture, infrastructure, property, and economy. Three major floods occurred in the last decade in 2010, 2011, and 2012, where the 2010 floods caused inundation of over 70,000 km^2^ and affected around 900,000 households^6^. The most recent’ 2022 Pakistan floods’ impacted approximately 33 million people, killing nearly 1500 people, and destroying critical infrastructure, agricultural land, and properties. In August 2022 Balochistan province of Pakistan received 590% more rainfall than normal while Sindh received 726% more rainfall than average for this month^7^. The World Weather Attribution group reports a 75% increase in heavy rainfall during the 60 days in the Indus River basin of Pakistan^8^.

The 2010, 2011, and 2022 floods in Pakistan affected approximately 55–60 million people with over 3500 deaths^6,9,10^. The floods in 2011 inundated over 21,000 km^2^ area, displaced 5.9 million people and damaged 1500 km of the road network, 382 km of railway tracks, 500 km^2^ of forests and over 16,000 km^2^ of agricultural land^6^. The 2012 floods inundated 13,157 km^2^ of the area in 22 districts of Pakistan damaging 2950 km^2^ of agricultural land, 1681 km of the road network, and 110 km of railway track^9^.

**Figure 1:**
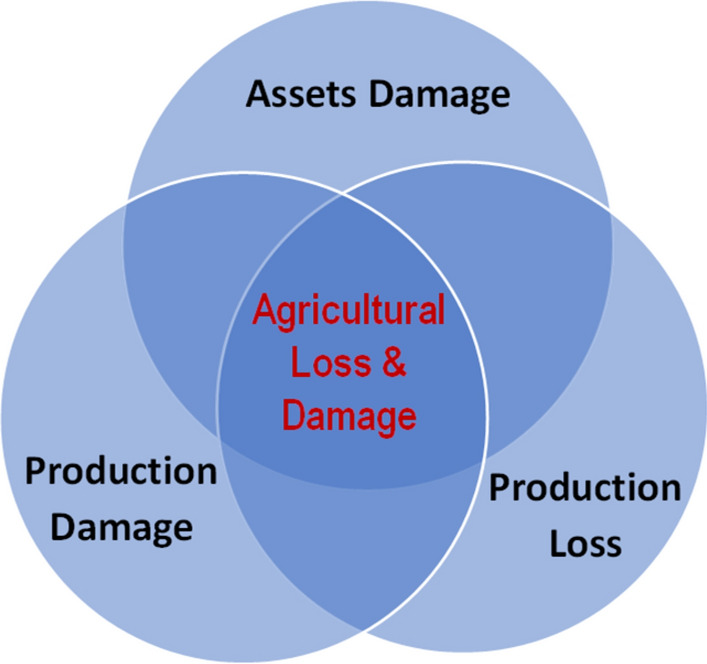
Key elements of agricultural Loss & Damage assessment.

The post-disaster damages assessment is extremely important in the context of global warming causing more frequent extreme events. The loss and damages (L&D) assessment is key to post-disaster recovery and rehabilitation under the conference of parties (COP) commitments^11^. Frameworks of L&D assessments enable evidence-based policies to reduce the risk of disaster and its impacts on various sectors. Adaptation of the L&D mechanism in the agriculture sector (Fig. 1) can help in monitoring and evaluating the economic impacts of disasters and support the compensation mechanisms. The damages are partial or complete destruction of physical assets while production losses refer to declines in the value of agricultural production resulting from a disaster. The production losses not only cause deficits in the income of impacted communities but may also result in near-term food shortages in the region and it is likely to slow down the post-disaster recovery if not properly managed.

Satellite remote sensing is a powerful tool for assessing L&D caused by natural disasters such as floods, hurricanes, and earthquakes. Particularly for flood disaster, Synthetic Aperture Radar (SAR) satellites data can efficiently map flood extent^12,13^ and optical satellites can be used to detect differences in vegetation cover before and after a flood to assess the damage caused by flooding^14,15^. Researchers have developed mathematical models to estimate flood losses based on the physical processes in a river basin and stage-damage relationship for different land use features for economic loss estimation^16^. As such, a novel index Disaster Vegetation Damage Index (DVDI) was also developed to map the post-flood crop-specific damage that occurred soon after the flood event^16^. In earlier work on crop loss assessments from Earth Observation, a Cyber Service system (RF-CLASS) was designed to estimate crop losses for insurance decision-making^17^. Likewise, the temporal differences in the satellite-derived Normalized Difference Vegetation Index (NDVI) have been applied for depicting changes in vegetation conditions to determine the impacts of extreme climate events on tropical secondary forest succession^18,19^. Another study developed an integrated approach by combining the NDVI, Land Surface Temperature (LST), and satellite-based estimates of rainfall to characterize vegetation moisture stress in land covers including forest, shrubland, agriculture and grass^20,21^.

Building on earlier work on L&D assessment and accommodating the recent advances in satellite remote sensing, we developed an approach for evaluating crop-specific post-disaster production losses, in the severely affected Sindh province of Pakistan. The tools and methodologies developed in this study will support evidence-based policies for food security and resilient reconstruction. This will also contribute to standardise reporting on climate change-related losses.

## Materials and methods

### Description of the study area

**Figure 2:**
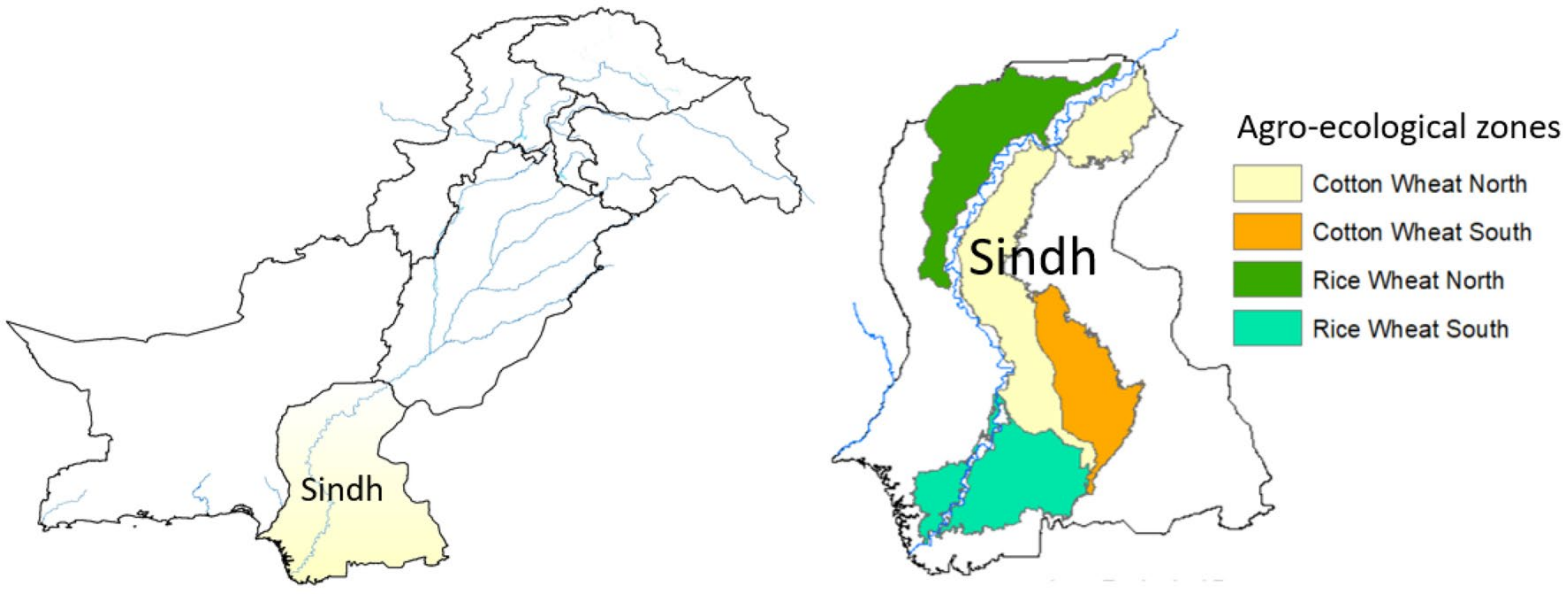
Location map of the study area and crop production zones in the Sindh Province of Pakistan, the maps were produced using the ArcGIS 10.7 (www.esri.com).

The study area comprises the Sindh province of Pakistan (Fig. 2), with a total land area of ~ 14.1 million ha, of which ~ 35% (4.9 million ha) is agricultural land. Approximately one-fourth of the national economy comes from Sindh, of which 17% is contributed by the agriculture sector. The agricultural lands are irrigated by Indus Basin Irrigation System to grow various crops in the province. The major crops in the study area include cereal crops (wheat and rice), industrial crops (cotton and sugarcane), fruit crops (mango and guava), and horticultural crops (chilli, tomato, and sunflower). The climate of Sindh is Subtropical, with hot summers (May–August) and cold winters (December–January). Temperature ranges from 2 °C in winter to 46 °C in summer. Mean annual rainfall is about 150–180 mm but most of this amount falls during the monsoon season (July–September).

### Remote sensing and ground-based rainfall estimates

The estimates of incidental rainfall were derived from the Integrated Multi-satellitE Retrievals for GPM (IMERG), GPM_L3/IMERG_V06, which is based on the unified algorithm combining data to generate a multi-satellite precipitation product ^22^. The hourly rainfall product was used for spatially contiguous rainfall estimates as an alternative to scarcely distributed station data in the study area. The data were obtained and processed using the Google Earth Engine (GEE), a cloud computing platform, to derive daily estimates at a native spatial resolution of 0.1° (~ 1 km). The data was acquired from 1st August 2022 to 31st August 2022. Historic (1981–2010) and recent (August 2022) daily rainfall measurements from the ground stations (namely Mohenjo Daro, Sakran, Padidan, Larkana, Badin, Jacobabad, Nawabshah, and Rohri), in the study area, were acquired from Pakistan Meteorological Department (PMD). These data sets were used for relating the satellite-based rainfall estimates with ground observations. The daily rainfall estimates were also aggregated at the monthly interval to understand the spatial patterns of monthly accumulated rainfall during August 2022.

### Flood extent mapping

Flood extent was mapped using pre- and post-event C-band Synthetic Aperture Radar (SAR) images from Sentinel-1B. The calibrated and ortho-corrected Level-1 Ground Range Detected (GRD) images at 10 m spatial resolution were obtained and processed using the ‘COPERNICUS/S1_GRD’ collection available on the GEE. Two sets of SAR images were acquired from, (1) May to July 2022, and (2) 22nd August to 3rd September 2022. Due to the specular reflectance of C-band signals, the backscatter from flooded areas or water bodies is significantly lower compared to other non-water areas, this enables rapid extraction of the flooded area from the SAR images^23^. Meticulous analysis of the pre-and post-flood images was performed to apply a parametric thresholding approach by splitting the histogram into the target and background classes to create binary maps indicating flooded and non-flooded areas^24–26^. Later, the binary images were processed to generate a flood extent map for subsequent analysis.

### Delta NDVI analysis for changes in greenness and ﻿damage assessment

To assess the intensity and spatial patterns of the flood’s impact on in-season crops, pre and post-flood images of Sentinel-2 were assessed to estimate the loss in greenness. The NDVI (Eq. 1), a frequently used indicator of green biomass^27^, was calculated using the reflectance wavebands corresponding to the Red and Near Infra-Red portion of the electromagnetic spectrum. Changes in the NDVI are related to photosynthesis and leaf cell structure which indicates a plant’s vigour, greenness and productivity^28^

1 $$NDVI= \frac{{NIR}_{\sigma } - {Red}_{\sigma }}{{NIR}_{\sigma } +{ Red}_{\sigma }}$$

where, ${NIR}_{\sigma }$ denotes the NIR reflectance band and ${Red}_{\sigma }$ corresponds to the Red reflectance bands.

To analyze and understand the phenology patterns associated with crop development phases, the NDVI was derived from all the available satellite images over the study area. It was noted that greenness over the cropland rapidly increases during August indicating a critical crop development phase during this period. Therefore, to incorporate the phenological differences and to compensate for the noise in the data, maximum NDVI value composite images (22nd August–3rd September) were developed for 2021 and 2022. A delta NDVI image (Eq. 2) was derived from the pre and post-flood NDVI composite images.

2 $$Delta NDVI (Loss or Gain \left(\mathrm{\%}\right))= \frac{{NDVI}_{f} - {NDVI}_{n}}{{NDVI}_{n}} \times 100$$

where ${NDVI}_{f}$ and ${NDVI}_{n}$ represents post and pre-flood NDVI composite images, respectively.

This delta NDVI image measures the loss and damage to cropland biomass, where the negative values correspond to the intensity of damage to cropland greenness or biomass due to flood-water inundation or incidental rainfall, while positive values show normal conditions. The resultant difference map was divided into eight classes representing extreme damage (< − 0.50], very severe damage (− 0.5 to − 0.4], severe damage (− 0.4 to − 0.3] moderate damage (− 0.3 to − 0.2], slight damage (− 0.2 to − 0.1], very slight damage (− 0.1 to 0], near normal (0–0.1) and normal (> 0.1). The loss and damage map was masked by the cropland mask derived from the ESA’s World Cover at 10 m resolution^29^. Later, to simplify the legend, the classes were lumped into three major classes representing severe loss (extreme damage and severe damage), mild loss (all other damage classes) and no loss (near normal and normal) for generating statistics at administrative levels. Then the percentage of loss or damage of cropland was aggregated at Tehsil and Districts level administrative boundaries for subsequent analysis. To distinguish the damage from continuous inundation and incidental rainfall, the loss and damage image was also masked by the flood extent map.

### Other data sources

The cropland, non-cropland and other land use land cover masks were developed from the ESA WorldCover 10 m 2020 v100 ‘© ESA WorldCover project 2020/Contains modified Copernicus Sentinel data (2020) processed by ESA WorldCover consortium’. The dataset is produced with 11 classes using Sentinel-1 and Sentinel-2 data^30^.

The population density data were obtained from the standardized global population distribution at 1 km spatial resolution provided by the LandScan™ Global Population Distribution Dataset (ORNL)^31^. Other data sources and data used for this study include administrative boundaries, Agriculture sector data^32^, field-based damage reporting data by the Provincial Disaster Management Authority, Sindh (PDMA), and Pakistan Crop statistics: Pakistan Bureau of Statistics.

### Analysis and aggregation of the data

Rigorous data analysis was performed to estimate and quantify loss and damages to cropland. The losses to agriculture and livestock were also converted to direct economic losses. For this, overlay analysis and zonal statistics of the flood extent map, NDVI loss and damage layer, incidental rainfall, and cropland masks were performed to map spatial extent and estimate area under flooded cropland, non-flooded cropland, agricultural damage due to incidental rain, severe and mild losses to the greenness of cropland. All the metrics were aggregated at district and tehsil (sub-district) boundaries to generate statistics for the administrative units. Percentage losses and damages were also calculated at the administrative units to understand the spatial spread of the flood’s impacts across the province. The loss and damage to the greenness of cropland were tabulated to rank the tehsil boundaries according to the majority of pixels belonging to the severity of the loss. Furthermore, maps were produced to portray the spatial distribution of flood extent and rainfall at the country level and detailed analysis and mapping were performed at the district and tehsil levels. This analysis framework is based on combining data from multiple satellites and ground-based information to assess and understand flood-induced damage and loss to cropland and livestock, and overall economic losses. The analysis was performed using the GEE and R.

## Results and discussion

### Above-normal rainfall in July and August across Pakistan

The 22nd Session of the South Asian Climate Outlook Forum (SACOF-22) was held in April 2022. According to the “Consensus Statement on the Seasonal Forecast over South Asia for the 2022 Southwest Monsoon Season”, the above-normal monsoon rainfall was projected in the South Asian region. An update of the consensus statement was also released in June 2022, reaffirming the extensive rainfall from July to September 2022. The national average rainfall in Pakistan for July 2022 was predominantly very high (+ 180%), with extremely high rains in Balochistan (+ 450%) and Sindh (+ 307%), the wettest for both in the last 62 years. The rainfall intensity was excessively increased in August 2022, the wettest ever August, with high above-average rainfall at the national level (+ 243%). Historically Sindh receives 150–180 mm of rainfall annually, which mostly comes in the monsoon season (July–September). Approximately six-fold rainfall was recorded in Balochistan (+ 590%) and Sindh received more than seven times the normal rainfall (+ 726%). The satellite-based rainfall estimates showed an extremely high intensity of monthly rainfall (~ 1000 mm) in the Sindh Province, nearly eight times its average rainfall in August (Fig. 1).

In August 2022, one of the most severe flash floods in the history of Pakistan was triggered due to above-normal monsoon rainfall in Balochistan, Sindh and southwest Punjab. The incidental and cascading rain-induced flooding started in July and both, rainfall intensity and flooding, progressively intensified in August which affected ~ 33 million people across the country. A sequence of flash floods started in July, and by the mid of August, one-third of the Sindh province was inundated by flood water. Moreover, the situation was further exacerbated by exceptionally high incidental rainfall (Fig. 4), for instance, 142 mm of rainfall was recorded in Naushahro Feroze District in a single day (11 August 2022), the highest monthly rainfall (1228 mm) in the province was recorded at the Padidan Town (Fig. 4) in the Naushahro Feroze District.

### Flood water inundation

**Figure 3:**
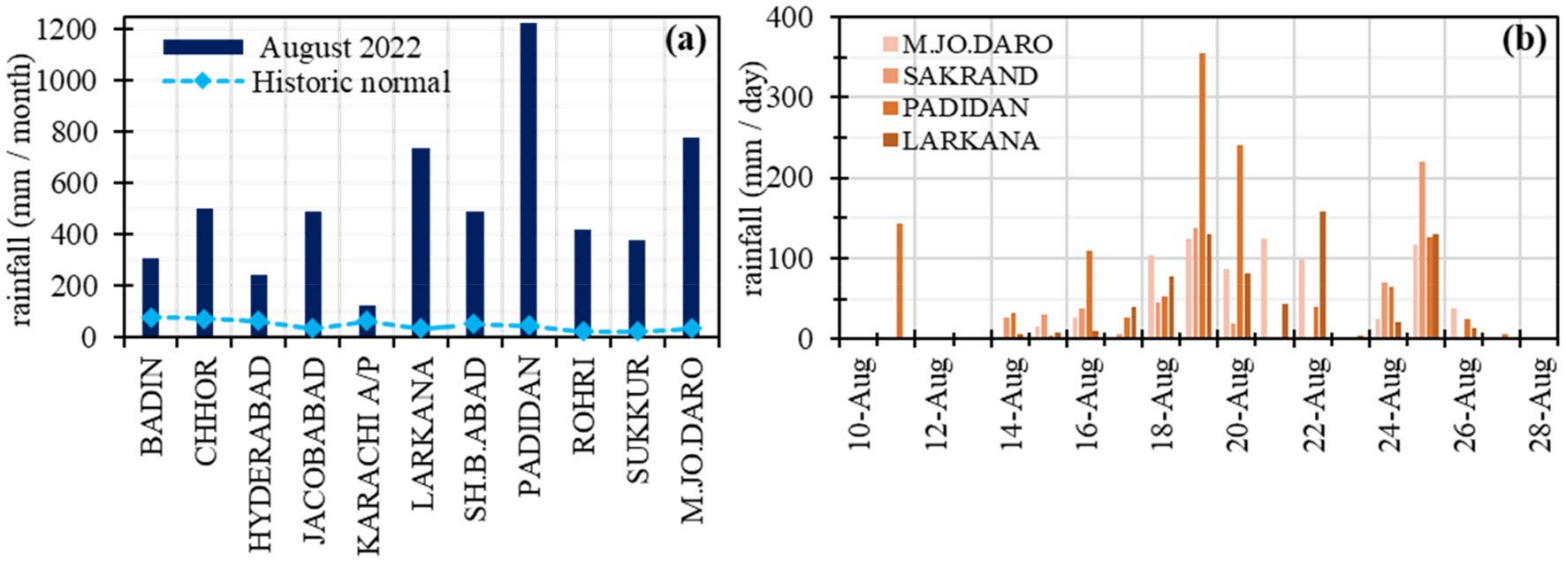
Station-based records of rainfall during August (**a**) Monthly average rainfall across different weather stations in Sindh during August 2022 and historic average in August (1981–2010), (**b**) daily rainfall patterns during August 2022 recorded in corresponding districts across Sindh. Source: Pakistan Meteorological Department (PMD).

**Figure 4:**
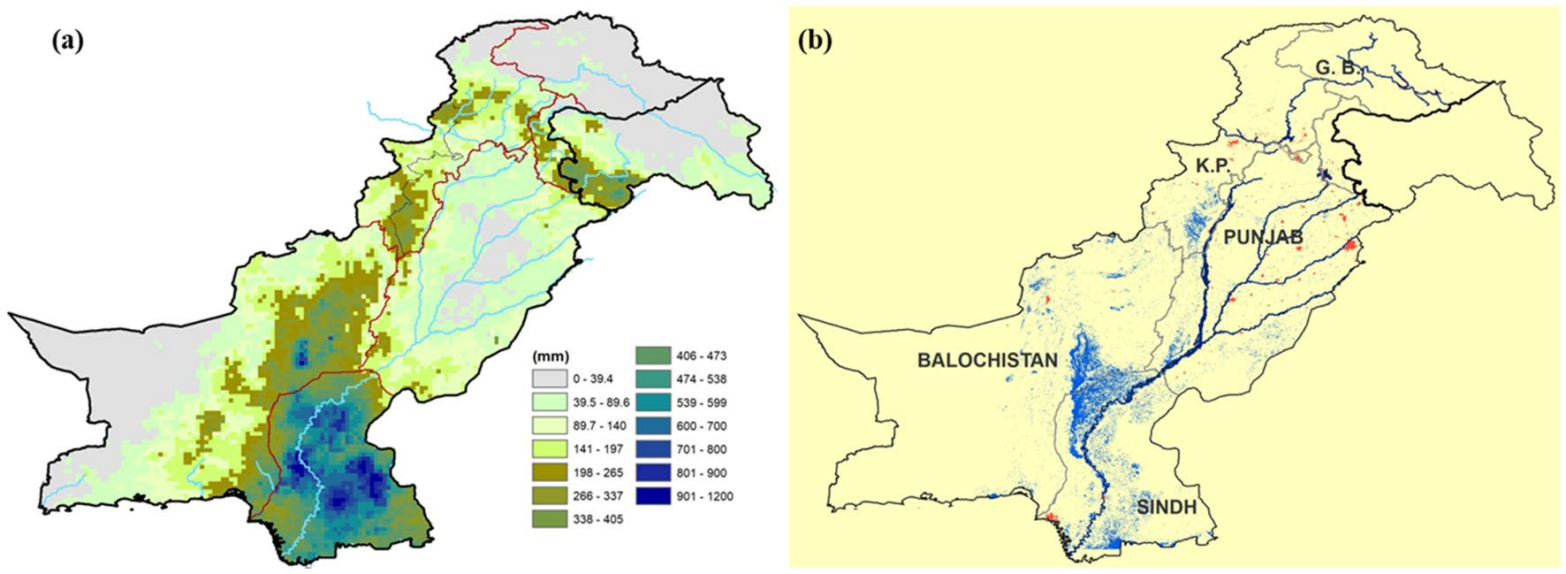
(**a**) Rainfall estimates for August 2022 in Pakistan (Source: GPM -IMERG satellite data), (**b**) Fig. 1: Flood extent in Pakistan on 28 August 2022 based on Sentinel-satellite data (Source: Sentinel-1 satellite data, the maps were produced using the ArcGIS 10.7 (www.esri.com).

**Figure 5:**
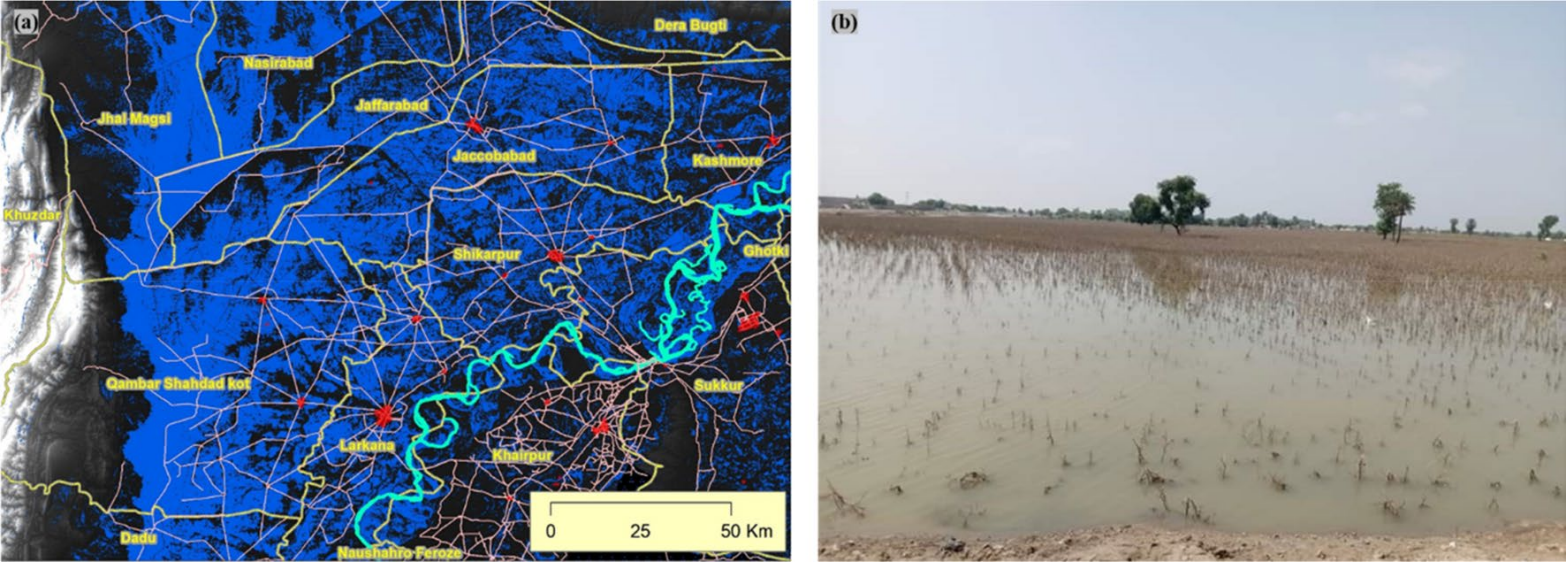
Flood water inundation (**a**) extent and patterns of flood-inundated areas in Jacobabad and Dera Murad Jamali on 28 August 2022 (**b**) ground condition information.

**Figure 6:**
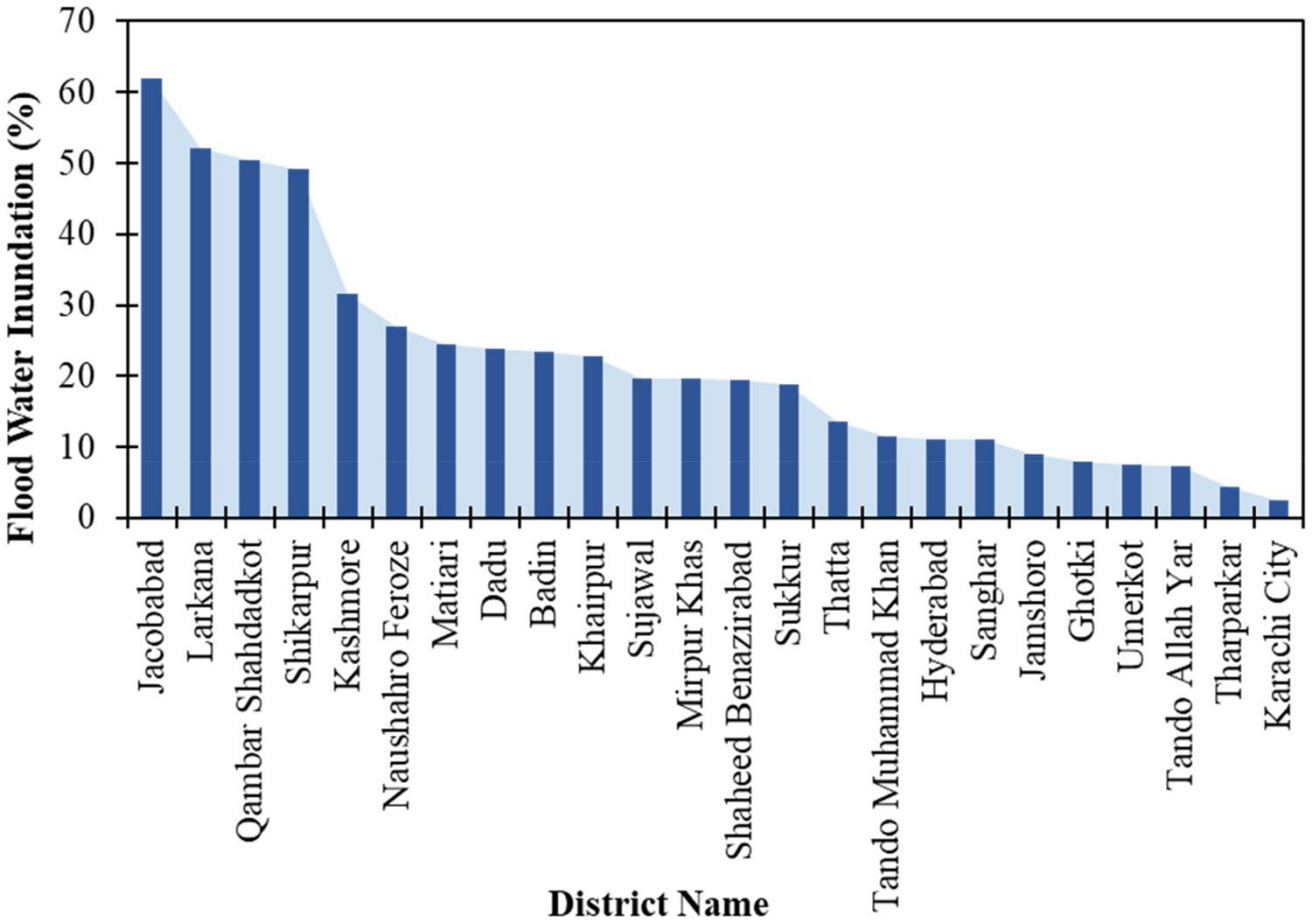
District-wise flood water inundation, ranked in descending order.

The flood extent mask for Pakistan (Fig. 4b) was derived from the Sentinel-1 SAR images. Detailed and aggregated flood maps were also produced for effective visualization and hotspot analysis along with population intensity maps. Nearly one-fifth (18%, 2.5 million ha) of the total land area of the province was waterlogged due to rain-induced flooding. District-level assessment of flood water inundation indicated four severely affected districts (Figs. 3, 4, 5) including Jacobabad, Larkana, Qambar Shahdad Kot, and Shikarpur, where the flooded area covered around half of the district (Figs. 4, 6). Due to the hill torrents in the Northeastern mountain ranges of Balochistan, the foothill areas in Balochistan and the north-western part of Sindh were severely affected by flash floods (Figs. 5, 6). The situation was exacerbated by the heavy rainfall in the affected areas which included Jacobabad, Kashmore, Larkana, and Shikarpur (Fig. 6). The district where the flood water inundated around one-fourth of the district included Kashmore (31.54%), Naushahro Feroze (27.05%), Matiari (24.57%), Dadu (23.82%), Badin (23.41%), and Khairpur (22.87%). Analysis of the flood extent map and cropland mask from the ESA WorldCover 10 m v100 showed that ~ 1.1 million ha of the cropland area was inundated in the Sindh Province.

A visual comparison of the rainfall intensity patterns (Fig. 4a) and flood extent map (Fig. 4b) indicates two distinct regions, North West of Sindh and North Eastern Balochistan, were severely affected by flood-inundated water (Fig. 5). These foothill regions lie in arid zones and are characterised by a flash floods and/or flood water inundation. The flood water inundated the foothill area and emerged as a 100 km inland water lake. Whereas central Sindh experienced the highest intensity of incidental rainfall, the amount of inundated water is comparatively less. It was also noted that the waterlogged area continued to expand through the increased water discharge at the Guddu barrage and rainwater coming through the western mountain region of Balochistan province. This caused flooding around the Indus River due to which mobility in most parts of the province became limited and access was obstructed.

### Damage to cropland

Sindh’s land area is ~ 14 million hectares of which ~ 35% (4.9 million ha) is used for agricultural purposes. Nationally, Sindh provides a significant proportion of summer crops including rice (42%), cotton (23%), and sugarcane (31%). The 2022 floods in Pakistan damaged the standing in-season crops, and huge quantities of grain in the storage, livestock, and infrastructure. The province is a production pocket of 14 Kharif season vegetables, 15 different fruits including major summer fruits, 5 different condiments and many cereal crops. This investigation did not estimate the specific crop type damages, nonetheless, an estimation of major crop losses and their spatial distribution was evaluated and discussed.

**Figure 7:**
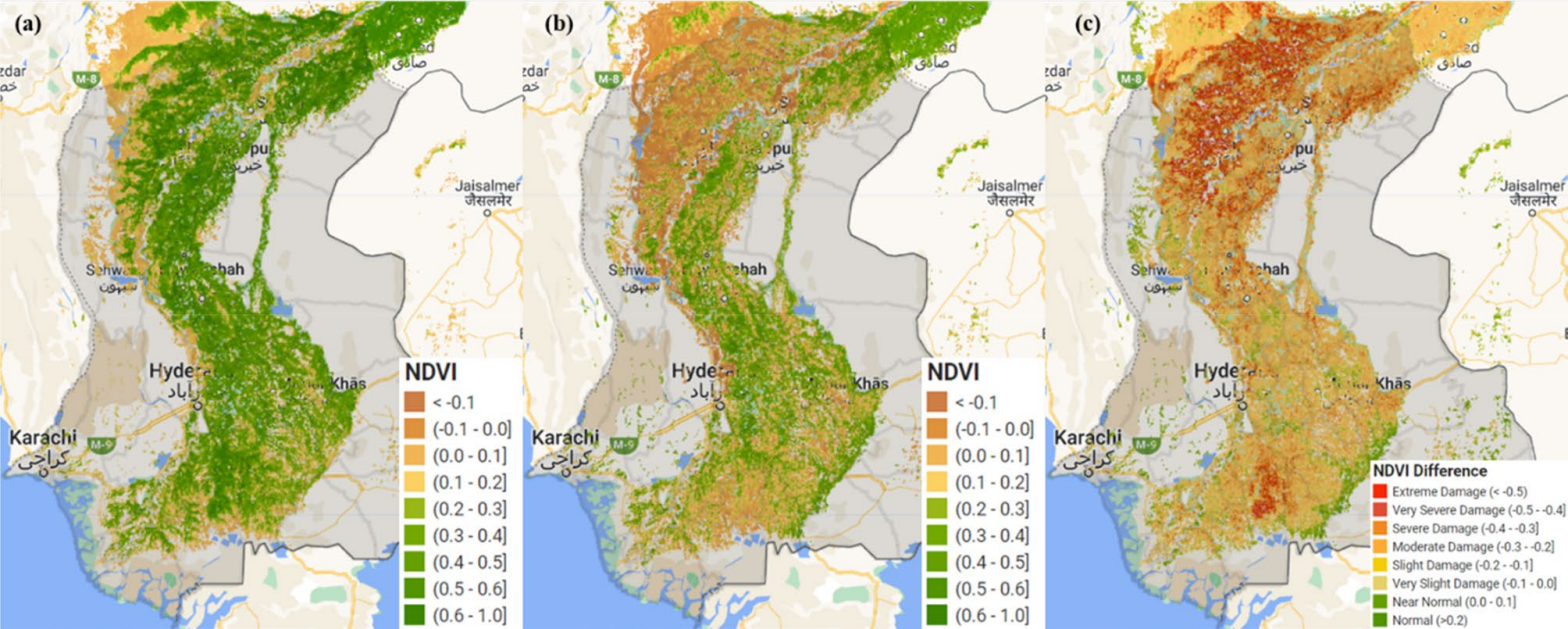
Loss and damage to cropland based on the NDVI difference (**a**) control or reference composite image, (**b**) composite image acquired during a flood event, (**c**) NDVI difference image indicating loss and damage to cropland. All the images were masked to cropland pixels. the maps were produced using the Google Earth Engine (https://earthengine.google.com).

The mapping of flood water inundation and crop damage assessment showed that ~ 2.9 million ha (18% of Sindh’s total area) was inundated and ~ 57% (2.8 million ha) of the cropland was affected (Fig. 7). The flood struck at a key phase of crop development—the initial stage of plant growth. Temporal patterns of NDVI were analysed to understand the crops’ phenology patterns from June 2021 to 31st August 2022 (Fig. A1). The results indicated the critical crop development stage during August, where the NDVI values rise with a high rate of change, before reaching maturity or maximum seasonal growth (Fig. A1). A flat curve in August 2022 implied severe loss of crop greenness and damage (Fig. A1 due to flood water inundation and incidental rainfall (Fig. 3). Apart from the plant damage due to incidental rainfall at critical crop development phases, flood-induced water logging in cropland can also trigger abiotic stress in crops^33^ by reducing light availability^34^, depletion of oxygen^35^, and changing the chemical properties of soil^36^. These alterations in the environment can significantly impede crop growth, vigour and yield^37–39^.

**Figure 8:**
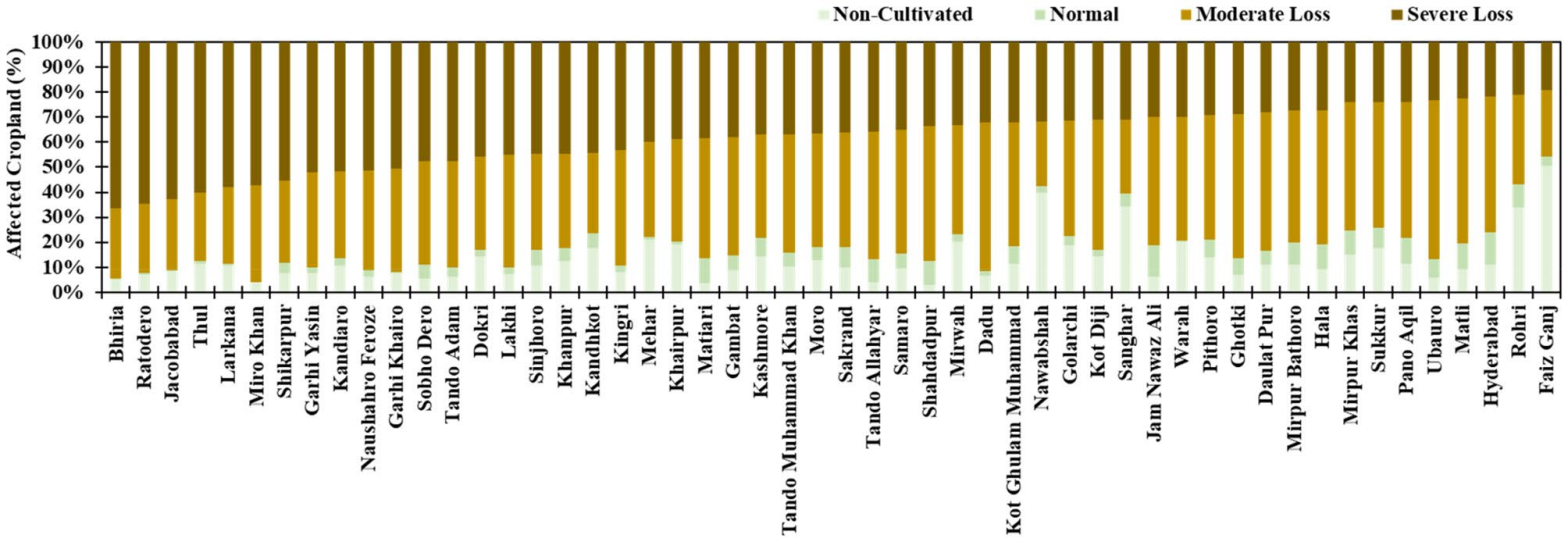
Tehsil-wise loss and damage to cropland derived from the NDVI difference map.

The delta NDVI maps were used to calculate the losses in greenness over the cropland. The results (Fig. 6c) were simplified into two classes indicating severe and moderate loss. It was noted that about ~ 36% of the cropland was severely affected while ~ 53% of the cropland was classified as moderately damaged. These losses included both the damages due to flood water inundation (one-third) and the remainder was directly damaged from torrential or incidental rainfall. The visual analysis of the NDVI changes (Fig. 6) along with rainfall and flood water inundation patterns suggested moderate damage to crops due to incidental rainfall but it was widely spread across the province. At the Tehsil level, the damage intensity ranges from ~ 90 to 50% (Fig. 8). According to the assessment, there were 50 Tehsils where the damage to the cropland was more than 50%. Nonetheless, it is important to discuss that the uncertainty in the NDVI-based analysis might prevail by changing the satellite images and composite period due to variation in crop development phases of the control image, emergence of herbaceous vegetation^39^, waterlogging tolerance of crops^38^, and death of the plants due to elongated flooded^38^. Moreover, in the mildly affected croplands, the production losses in yield could also vary with the duration of flooding and type and/or a variety of crops^40,41^.

Remote sensing-based vegetation indices have been used to derive indicators for crop development stages and impacts of flooding on crop growth conditions compared to pre-flood conditions^42–47^. Recently, a framework is developed for an integrated assessment of flood impacts on crop protection in the Yangtze River Basin^39^. However, this study investigated the post-flood damage at the end of the crop growing season by taking the difference in vegetation index between the normal year and flood-affected year^47^. Moreover, conventionally, flood impact studies neglect the effect of spatial–temporal variations and in-seasons crop damage assessment in severely flooded areas. This study developed the delta NDVI composite maps to determine the in-season crop loss by leveraging the power of GEE cloud computing and the availability of high temporal resolution of Sentinel-2 images.

**Figure 9:**
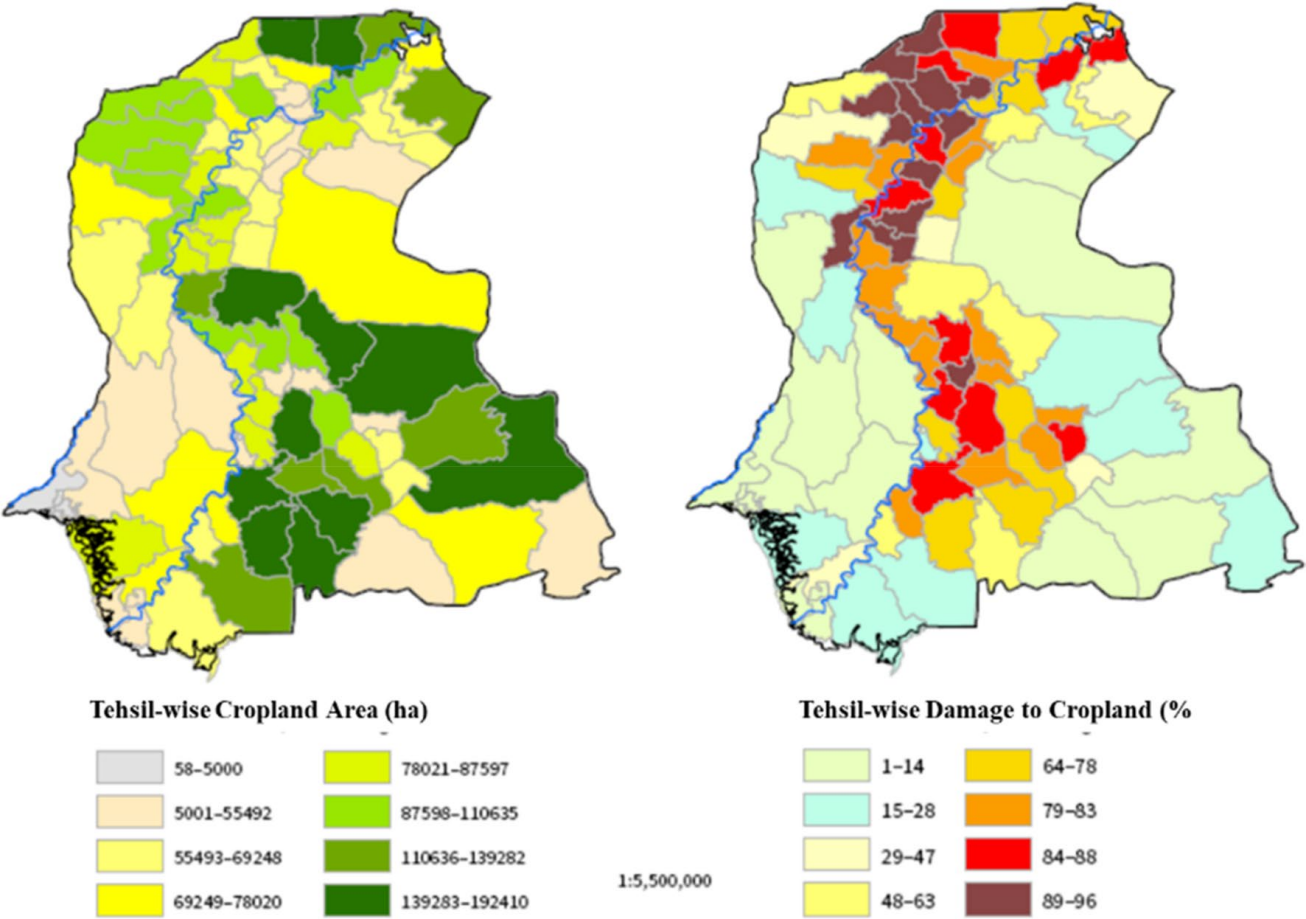
Cropland area and intensity of the damage to cropland due to flood at Tehsil level.

Figure A2 shows rice, cotton, and sugarcane are the three major commercial crops cultivated throughout Sindh in the Summer season (Kharif crops). The distribution patterns of these primary crops in the province in very significant (Fig. A2). Interestingly, each of these crops is affected differently, for instance, the rice crop is mostly affected by the water logged in the north-western part. It is estimated that ~ 80% of the rice crop production (~ 1.9 million tons of rice) is lost due to flooding (Figs. 2, 6, 9, A2). The rice production zone was severely waterlogged due to the flooding which can extinguish plants by limiting light availability and oxygen supply. Table 1 summarises the expected loss in crop production at the district level. On the other hand, the sugarcane crop is predominantly cultivated in the northeastern districts along the Indus River. There was lesser evidence of flood water logging in the sugarcane zone, probably due to the better drainage mechanism of this side of the river and partially due to the well-grown plant of the sugarcane crop. However, the dispersed population and flooding could generate an expected loss of 61% (10.5 million tons) of the crop by the end of the crop-growing season. It was important to note that the cotton crop zone was the least inundated, nonetheless, the loss of cotton crop was significantly high due to the incidental rainfall. The hotspot of rainfall intensity was exactly over the cotton crop zone which could have severely affected the sensitive plants of the cotton crop. The total loss in cotton crop production is expected to be 88% (3.5 million bales) because of flooding and continuously exceptionally heavy rainfall. Considering the commodity prices of summer 2022^48^, these three crops faced a direct loss of USD 1.30 billion (rice: USD 543 million, cotton: USD 485 million, sugarcane: USD 273 million).

**Table 1:** Expected production loss of rice, cotton and sugarcane crops in Sindh.

District	Loss of crop production		
	Rice	Cotton	Sugarcane
	metric tons	bales	metric tons
Badin	320,844	94,432	1,156,688
Dadu	56,547	31,112	100,850
Ghotki	77,620	519,113	1,378,575
Hyderabad	2321	35,872	231,635
Jacobabad	255,803	nr	3158
Jamshoro		42,134	1,989
Kambar-Shahdadkot	181,561	nr	
Karachi		nr	
Kashmor	206,507	nr	
Khairpur	6,077	390,125	286,166
Larkana	335,360	nr	31,870
Matiari		199,418	805,470
Mirpurkhas	8770	196,632	917,677
Naushahro Feroze	24,261	172,345	1,040,376
Sanghar	7,561	634,346	380,237
Shaheed Benazirabad	23,406	301,673	1,298,054
Shikarpur	299,021	nr	18,919
Sujawal		nr	
Sukkur	6846	144,537	140,323
Tando Muhammad Khan	30,199	13,187	1,166,367
Tando Allahyar		124,321	1,056,866
Thar		873	4567
Thatta	49,470	16,942	424,746
Umerkot		189,647	42,261
Loss of production	1,892,172	3,106,709	10,486,793
Total production in Sindh Province (2020)	2,374,300	3,523,400	17,233,830
Loss of production (%)	80	88	61

These estimates of production losses could be exacerbated while considering the abandoned or unattended agricultural fields due to dislocated and suffering population. It would be difficult for small farmers to immediately recover from loss which might trigger a household transition from on-farm to off-form work for livelihood^49,50^. Apart from the losses and damages that can be termed economic, the floods have caused more non-economic losses and damages that are difficult to assess in economic terms. These noneconomic losses may be more significant for developing countries for which such losses should become a central aspect of climate change policy. When land is lost or rendered unsuitable for agriculture the rich landowners may suffer greater losses in economic terms.

The rapid assessment loss estimates through this approach closely match with the detailed Post-Disaster Needs Assessment^51^ which reports cotton as the most affected crop followed by rice and sugarcane. It has been observed that rapid estimates of disaster losses for major events are more accurate than the small-scale local disaster loss events^51^. The true economic costs of climate change are difficult to quantify due to a lack of reliable data. The costs of climate change are often long-term and uncertain, making it difficult to accurately measure. The losses related to long-term impacts like reduction of soil productivity and indirect costs such as displacement and disruption to local economies may be difficult to quantify, while the intangible costs associated with loss and damage (such as emotional distress, loss of culture and traditional knowledge, etc.) cannot be measured.

## Conclusion and recommendations

Numerous efforts are being made to reduce agriculture losses and damage to assets ahead of impending hazards by providing forecasts and early warning. However, the frequency of natural disasters has increased due to climate change and exacerbates food security issues across the globe, especially in developing economies. Therefore, apart from precise predictions of changing weather, e.g., floods, erratic and above-normal rainfall, and droughts; it is required to quickly evaluate the impacts of disasters to avoid their cascading effects. This study presented a multi-satellite/sensor-based framework to quantify the impacts of flooding, water logging and torrential/or incidental rainfall on in-season crops in the Sindh province of Pakistan. The province provides a significant portion of major crop production in the country including wheat (16%), rice (42%), cotton (23%), sugarcane (31%), vegetables and other horticulture crops.

It is equally important to translate climate-induced losses into the economic cost to quantify the benefits of the adaptation strategies. This can enable the implementation of resource allocation for a holistic climate change adaptation in public expenditure reporting. The information derived from this analytical framework can be incorporated into L&D assessment for informed decision-making not only during the catastrophe and rehabilitation of the affected people, but it can also help to timely plan for the economic losses and looming crisis of food security in the country. The framework could be extended and further developed to integrate economic losses of crop damage, loss of agriculture infrastructure and equipment and disruption in supplies and services. It is difficult to accurately attribute losses and damages to climate change due to the complexity of the climate and governance systems resulting in cumulative losses. Also, the costs of climate change are often long-term and uncertain, making it difficult to accurately measure.

## Supplementary Information

Supplementary Figures.

## Data Availability

The satellite datasets were processed and obtained using the cloud computing platform, Google Earth Engine. All the datasets are available. The datasets analysed during the current study are available from the corresponding author upon reasonable request.
