# Supplementary material for: A framework for multi-sensor satellite data to evaluate crop production losses: the case study of 2022 Pakistan floods
Source: Sci Rep. 2023 Mar 14;13:4240. doi: 10.1038/s41598-023-30347-y (PMC10015072; doi:10.1038/s41598-023-30347-y)
Supplement: Supplementary file 1 — Supplementary Figures. [file 41598_2023_30347_MOESM1_ESM.docx]

**A Framework of Multi-Sensor Satellite Data to Evaluate Impacts of the 2022 Pakistan Floods on Crop Production Losses in Sindh**

Faisal Mueen Qamer^1,*,^ **^¶^**, Sawaid Abbas^2,3,**,^ **^¶^**, Bashir Ahmad^4^, Abid Hussain^1^, Aneel Salman^5^, Sher Muhammad^1^, Muhammad Nawaz^4^, Sravan Shrestha^1^, Bilal Iqbal^4^, Sunil Thapa^1^

^1^International Centre for Integrated Mountain Development (ICIMOD)

^2^Smart Sensing for Climate and Development, Center for Geographical Information System, University of the Punjab, Lahore, Pakistan

^3^Department of Land Surveying and Geo-Informatics, The Hong Kong Polytechnic University, Hong Kong

^4^Pakistan Agricultural Research Council (PARC), Pakistan

^5^Islamabad Policy Research Institute (IPRI), Pakistan

Correspondence: [sawaid.abbas@gmail.com](mailto:sawaid.abbas@gmail.com) (S.A) , [Faisal.Qamer@icimod.org](mailto:Faisal.Qamer@icimod.org) (FMQ)

^¶^These authors contributed equally to this work, shared the first authorship


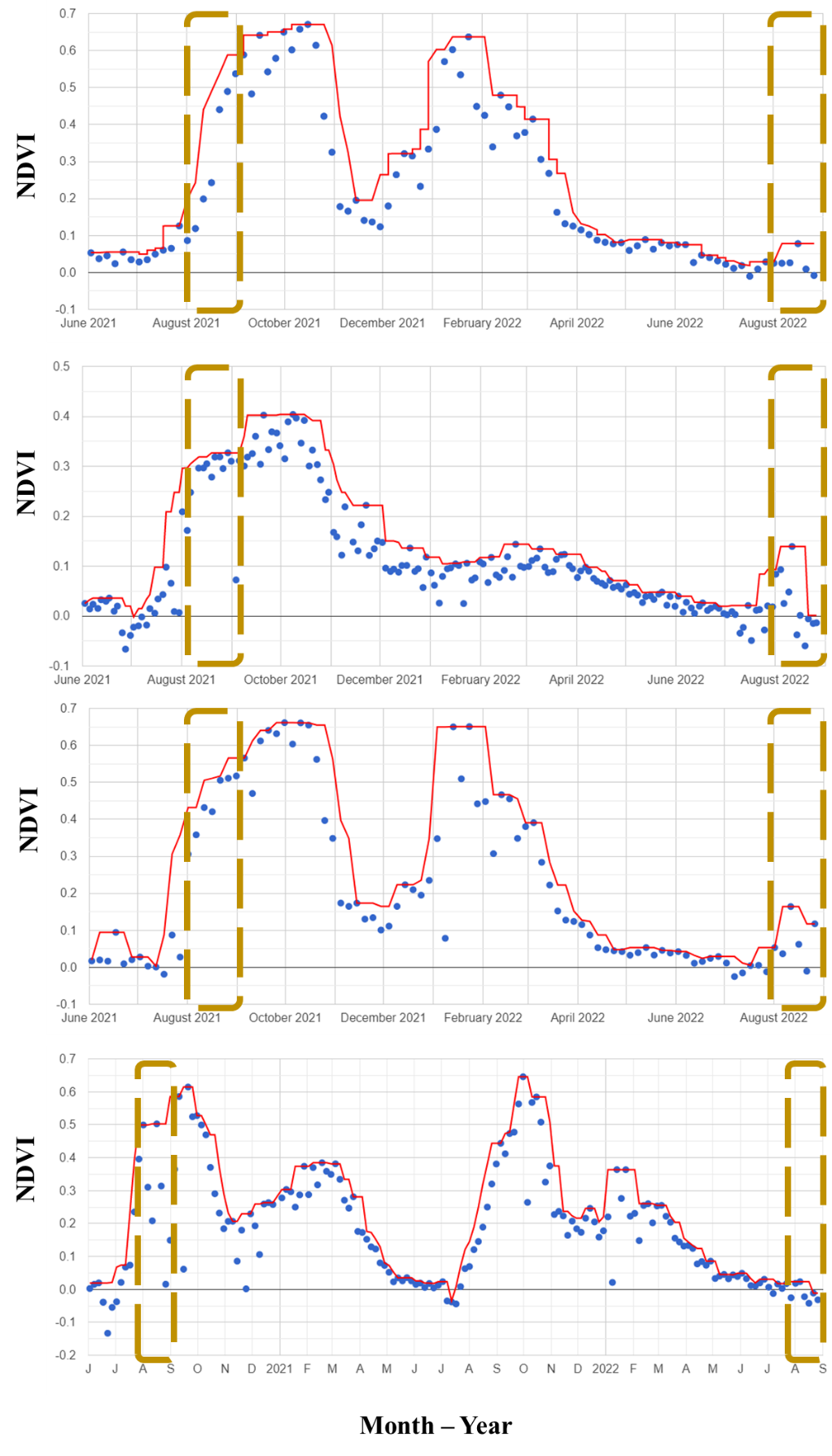


**Fig. A1.** Temporal patterns of NDVI indicating crop development phases from June 2021 to 31^st^ August 2022. Rapid growth during August is critically important for crop development. Loss of crop development is evident in August 2022.

#
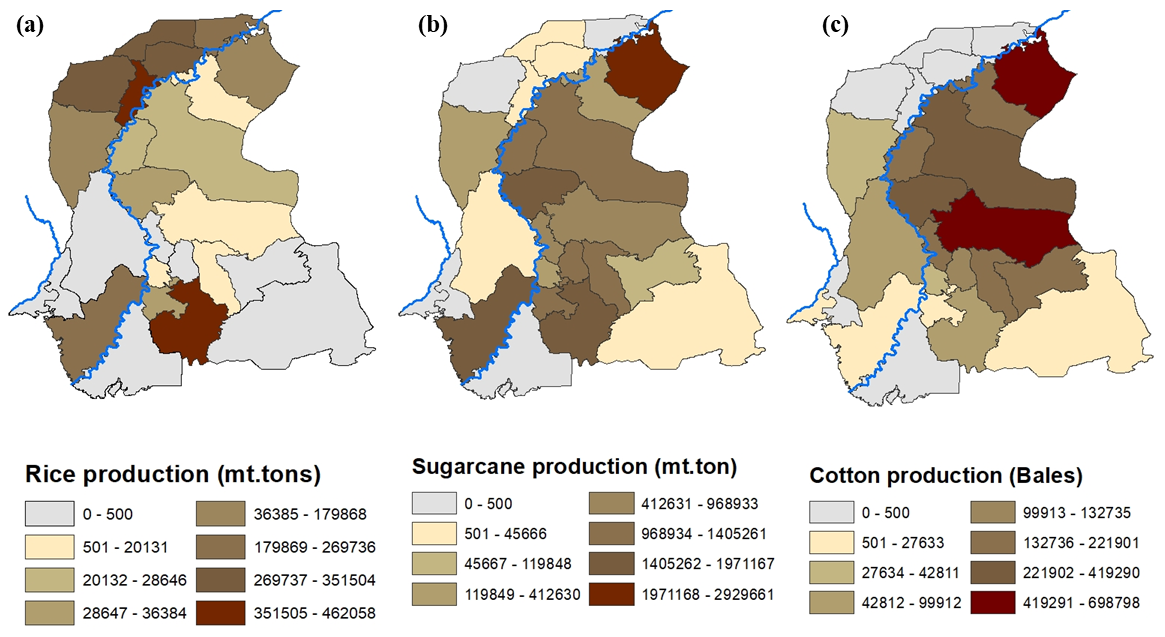


**Fig. A2.** Spatial patterns of production the three major crops in Sindh Province. (a) Rice production patterns, (b) Sugarcane production patterns, and (cotton production patterns), the maps were produced using the ArcGIS 10.7 ([www.esri.com](http://www.esri.com))
